# Supplementary figures and images for: Efficient and flexible Integration of variant characteristics in rare variant association studies using integrated nested Laplace approximation
Source: PLoS Comput Biol. 2021 Feb 19;17(2):e1007784. doi: 10.1371/journal.pcbi.1007784 (PMC7928502; doi:10.1371/journal.pcbi.1007784)

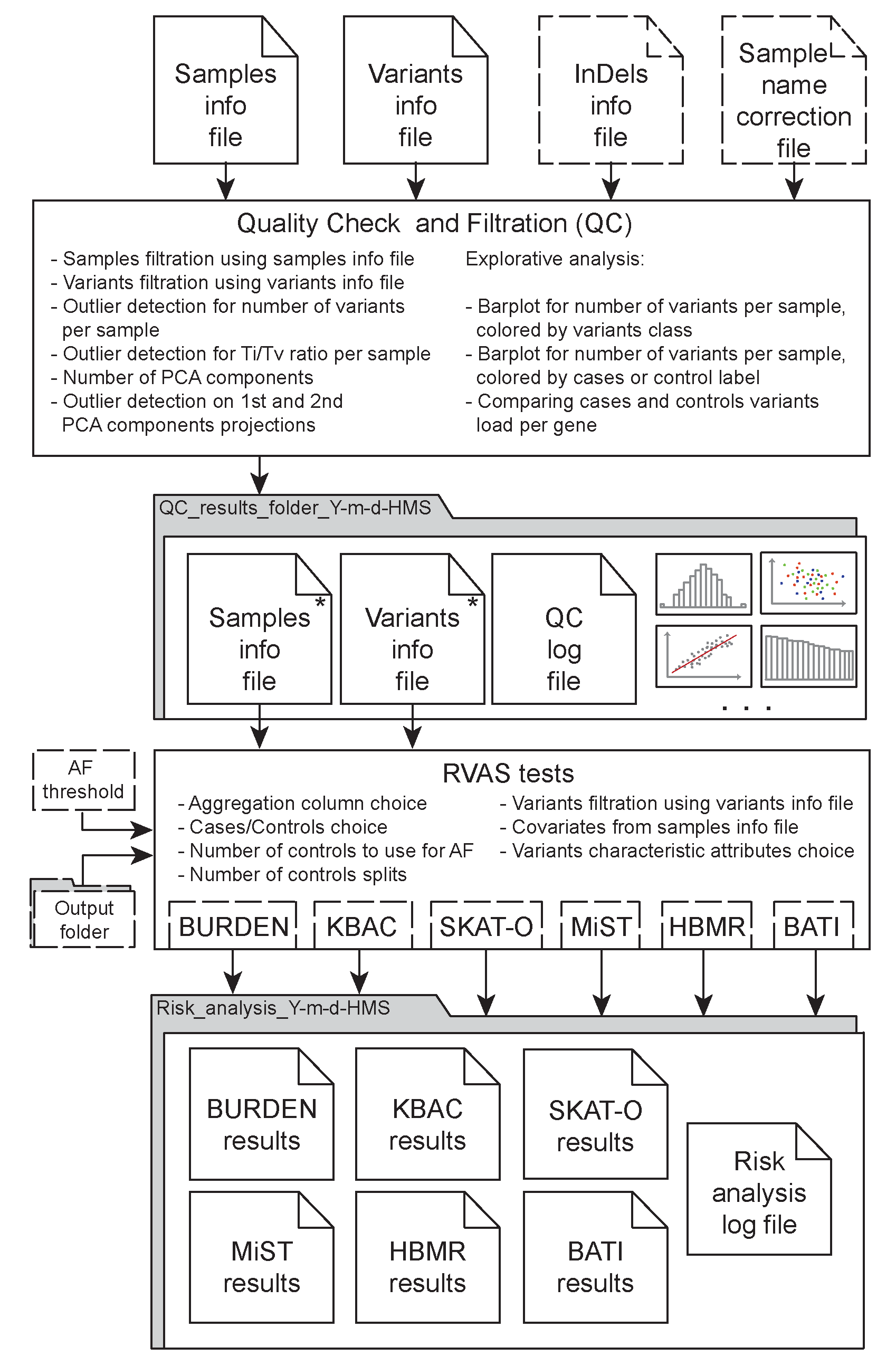

Supplement: S1 Fig — (TIF) [file pcbi.1007784.s001.tif]

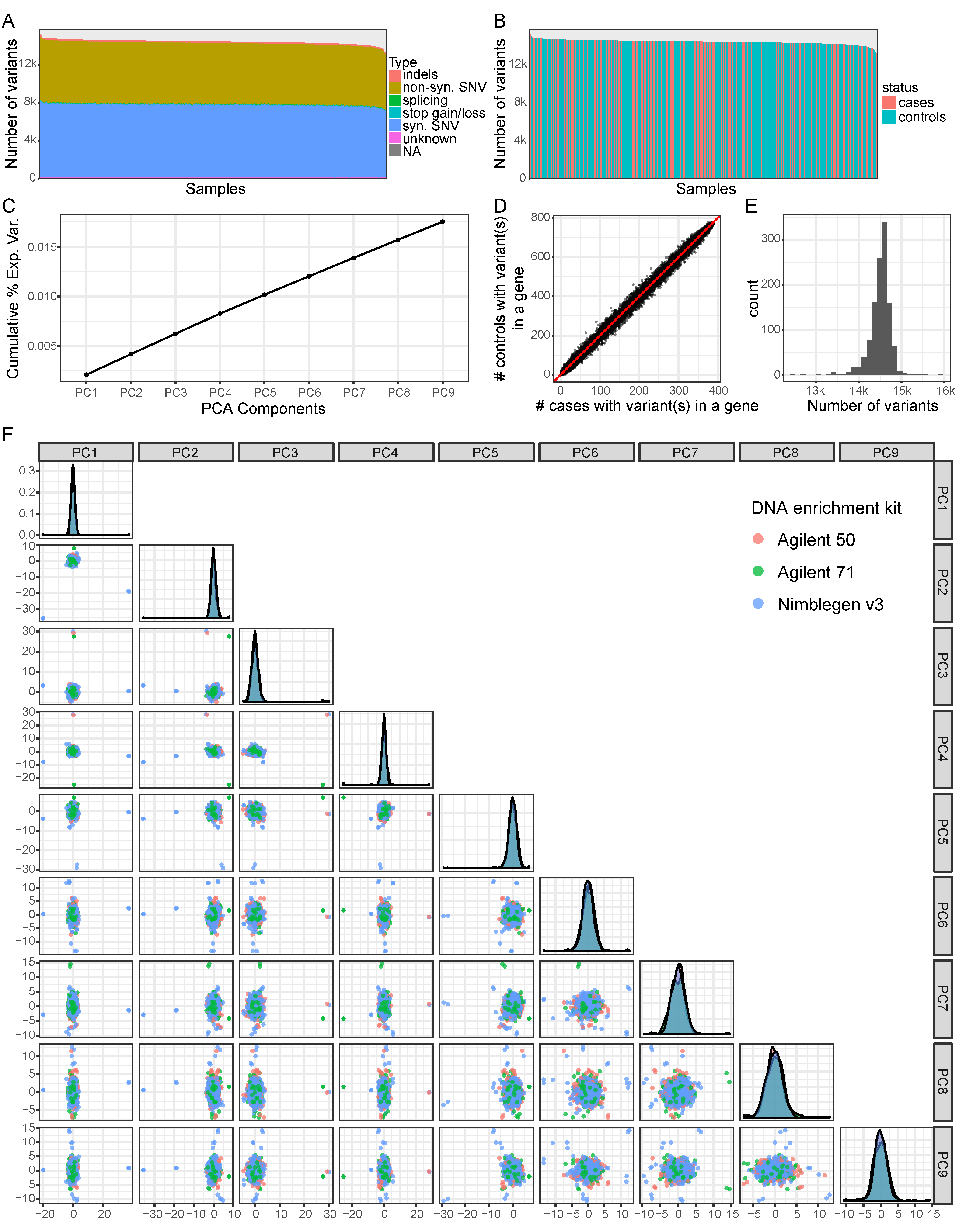

Supplement: S2 Fig — The 1,167 samples coming from Iberian population are used for benchmarking. rwGWAS QC showed the following QC statistics (A) Bar-plot for number of variants per sample, colored by variant type, (B) Barplot for number of variants per sample, colored by random assignment to cases (~1/3) or controls (~2/3), (C) Percentage of explained variance on first 9 PCA components, (D) Number of variants per gene in cases (x-Axis) and controls (y-axis). Each dot is one gene, while the red line shows the ratio of the number of cases and controls (1:2), (E) Histogram for number of mutations per sample, and (F) Projection on first 10 PCA components. Samples are colored by the center that performed the sequencing. (TIF) [file pcbi.1007784.s002.tif]

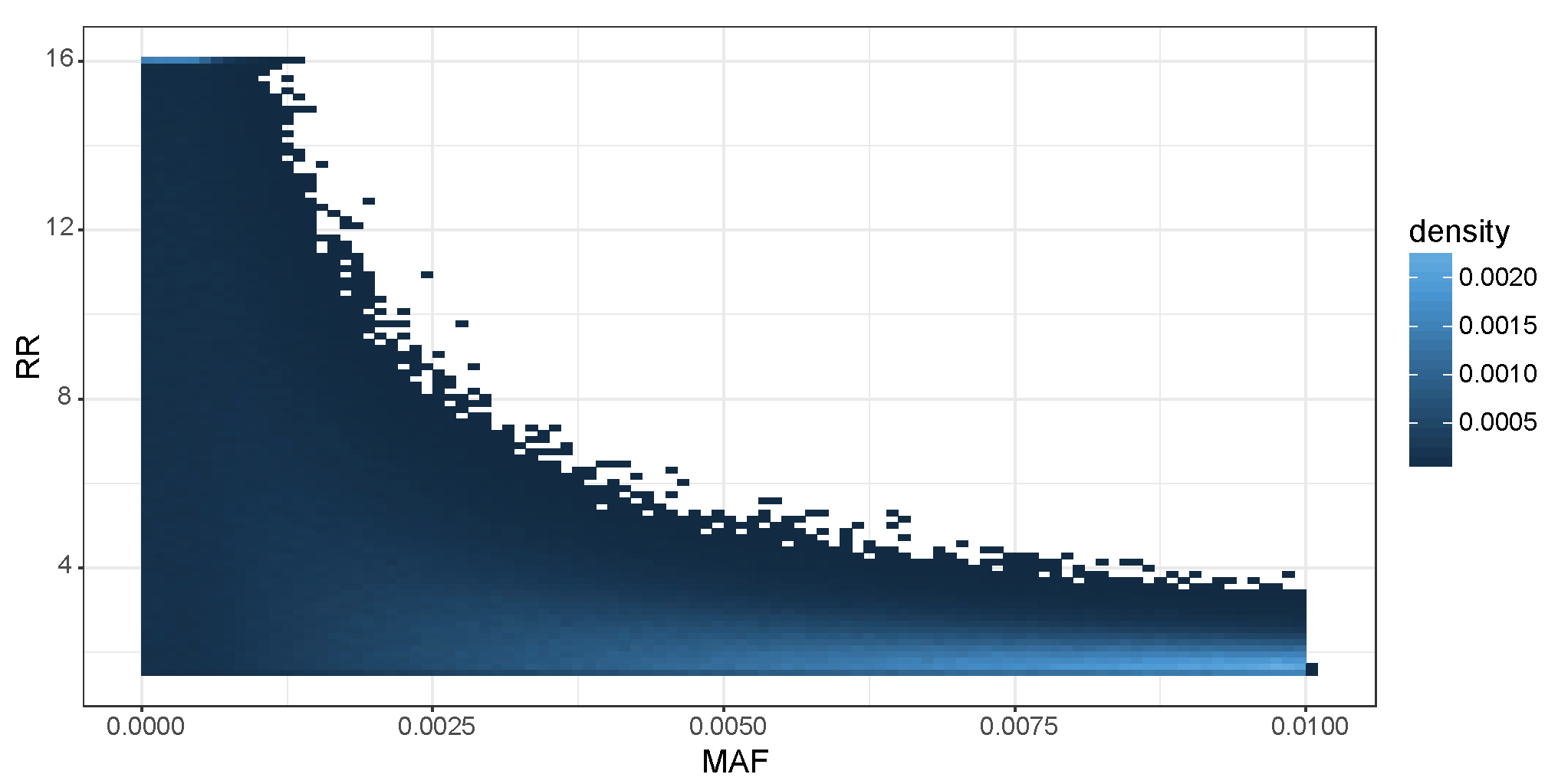

Supplement: S3 Fig — (TIF) [file pcbi.1007784.s003.tif]

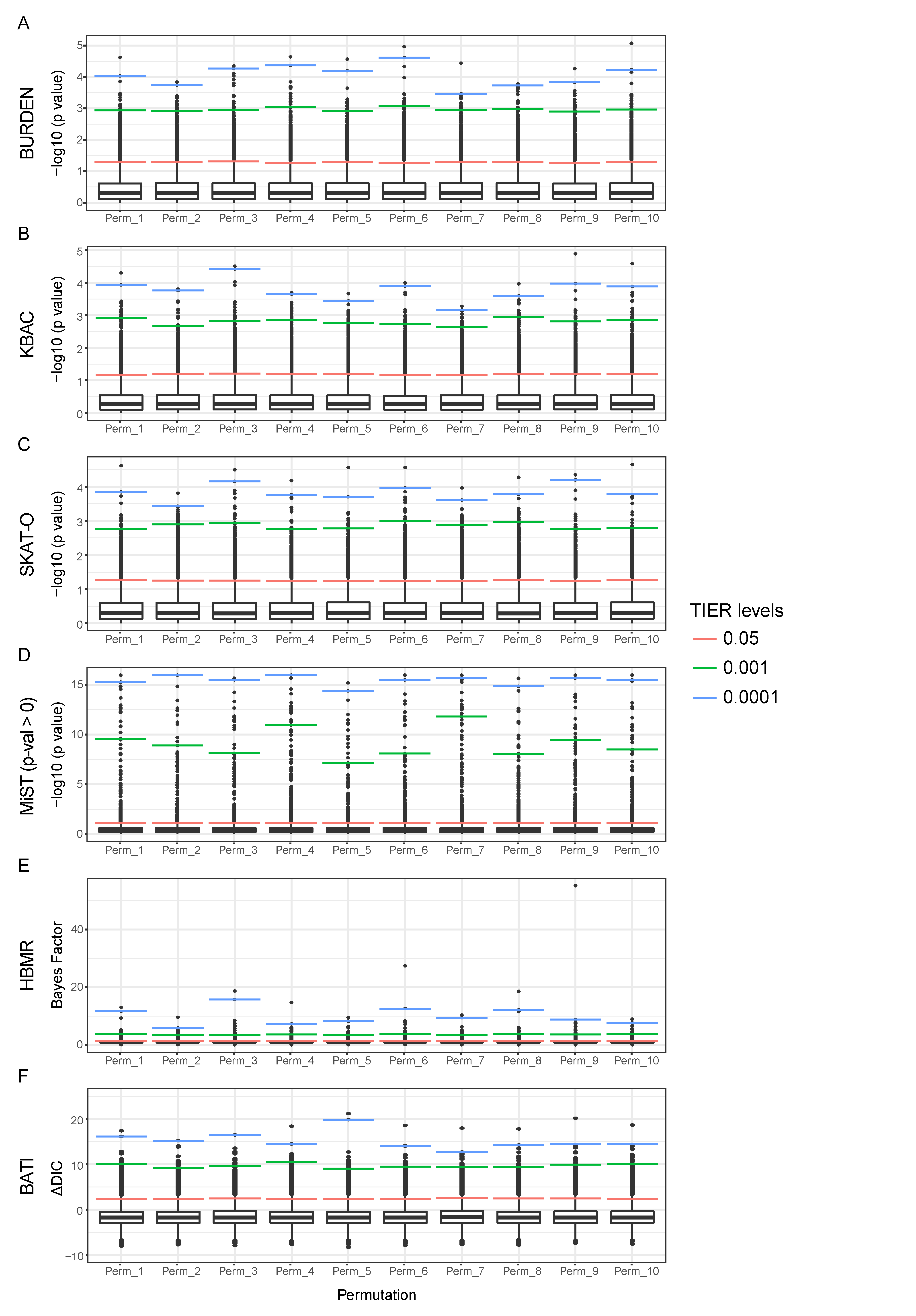

Supplement: S4 Fig — In 10 random splits of the 1000GP dataset into cases and controls, three commonly used significance levels thresholds (TIERs) are estimated: 0.05, 0.001 and 0.0001. (TIF) [file pcbi.1007784.s004.tif]

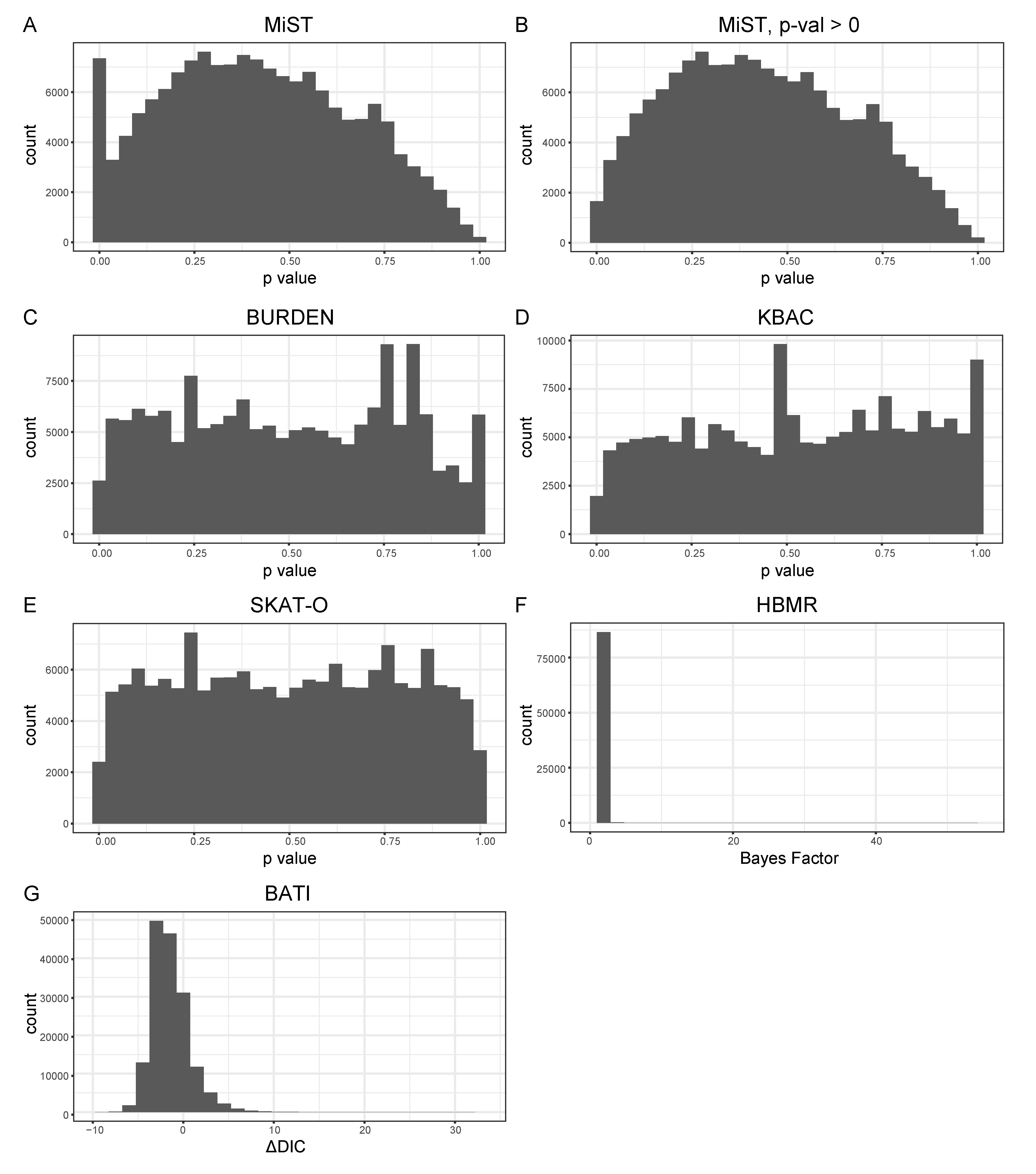

Supplement: S5 Fig — P-value, Bayes Factor and ΔDIC distributions. For each RVAS test we created distribution of p-values/Bayes Factor/ Δ DIC with randomly assigned cases and controls in 1000GP dataset. In panels are shown distributions for (A) MiST, (B) MiST with p values larger than zero, (C) Burden, (D) KBAC, (E) SKAT-O, (F) HBMR, and (G) BATI RVAS test. (TIF) [file pcbi.1007784.s005.tif]

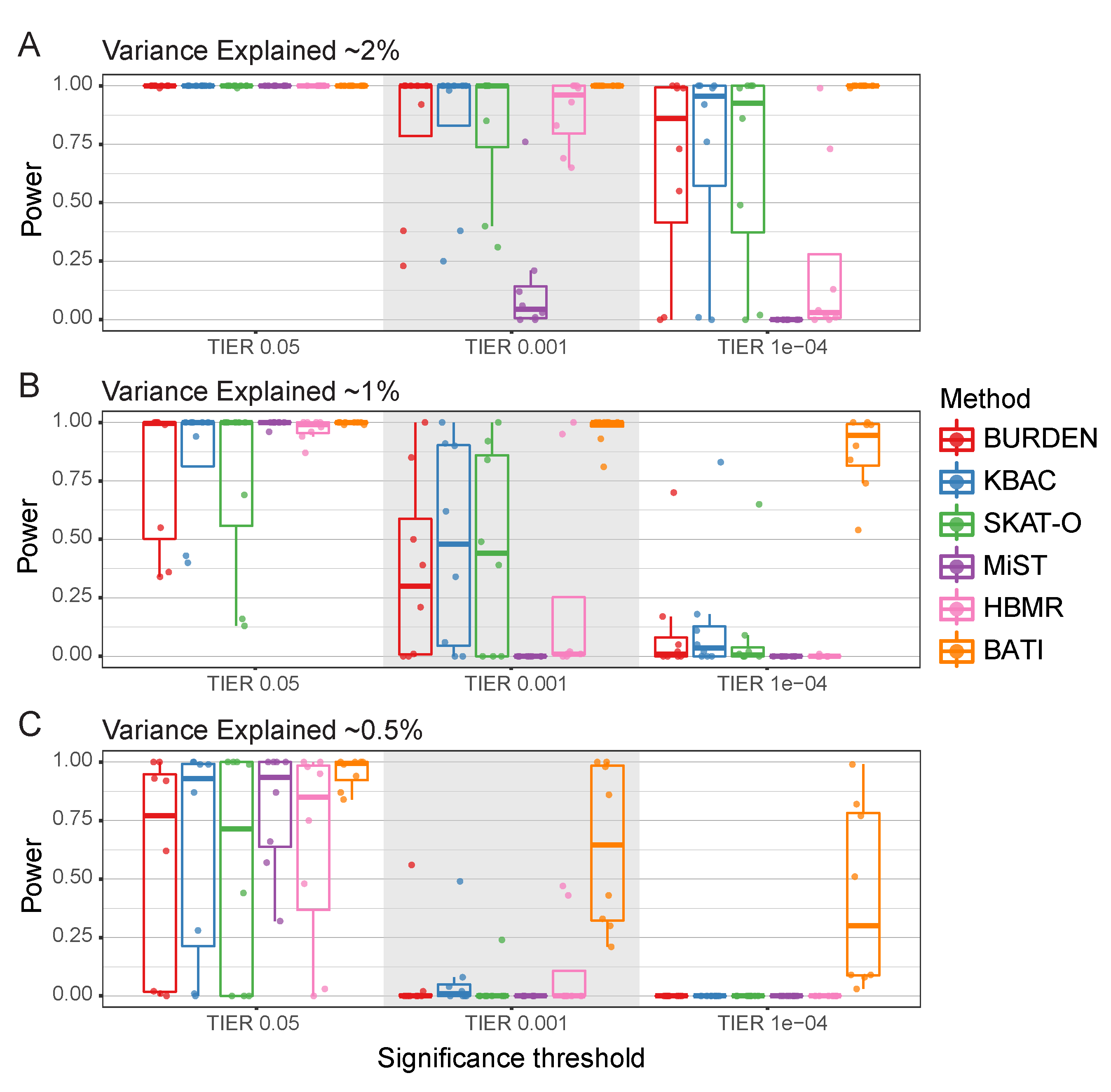

Supplement: S6 Fig — Each dot in the plots represents one of 8 risk genes, and y-axis values show the fraction of 100 simulations in which the gene was called as significant. Variance explained of the incorporated risk variants (A) ~2%, (B) ~1%, and (C) ~0.5%. (TIF) [file pcbi.1007784.s006.tif]

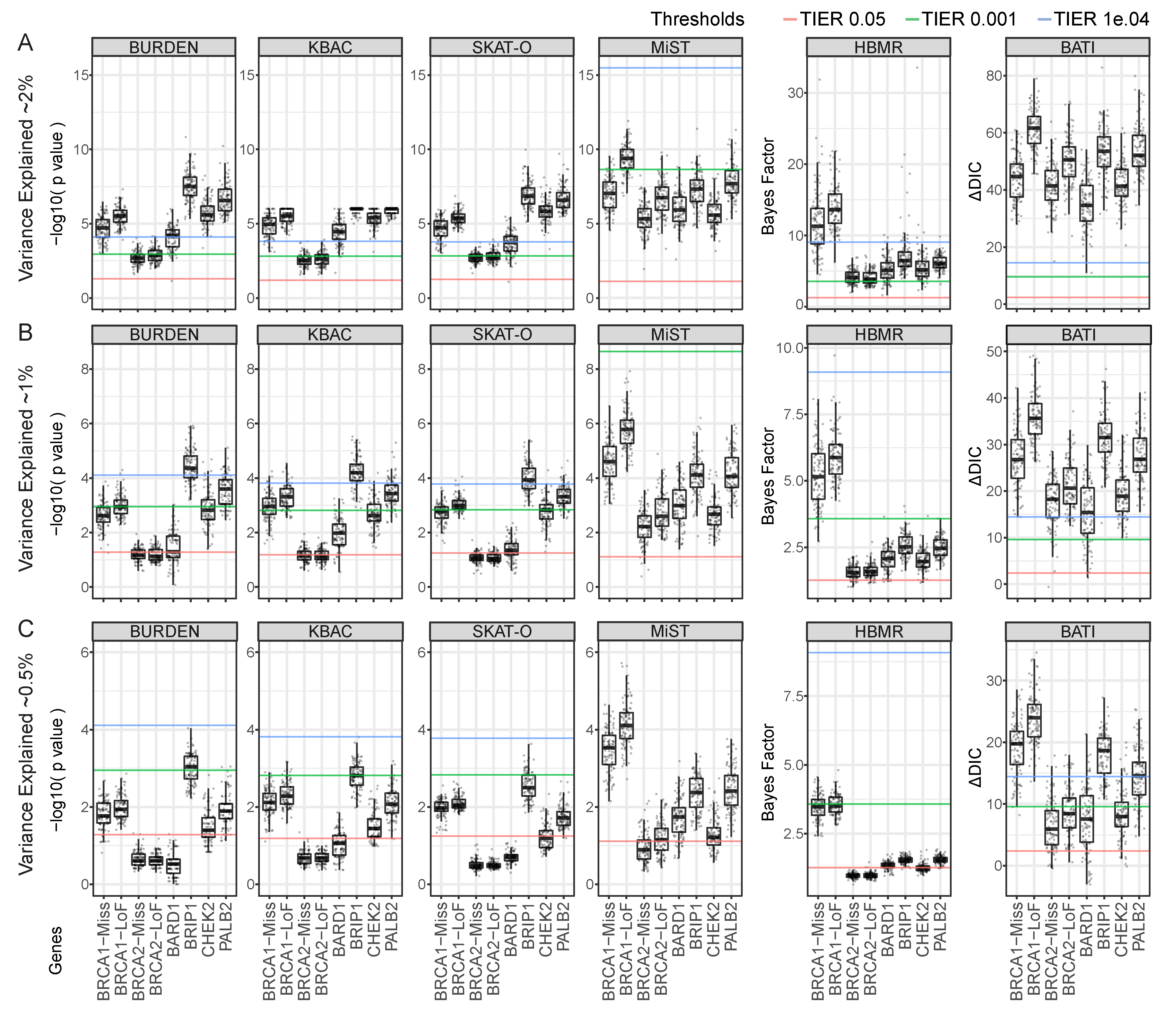

Supplement: S7 Fig — Rare variants annotated for increased breast cancer risk were simulated into 1000GP dataset with cases and controls randomly assigned. Results per gene for 6 methods (Burden, KBAC, SKAT-O, MiST, HBMR and BATI) are shown for (A) 2%, (B) 1%, and (C) 0.5% variance explained between cases and healthy controls. Due to using real SNVs in the simulation the real variance explained per gene fluctuates slightly around the targeted VE (see S2 Fig). Red, blue and green lines indicate relaxed, medium and strict TIER thresholds, respectively. (TIF) [file pcbi.1007784.s007.tif]

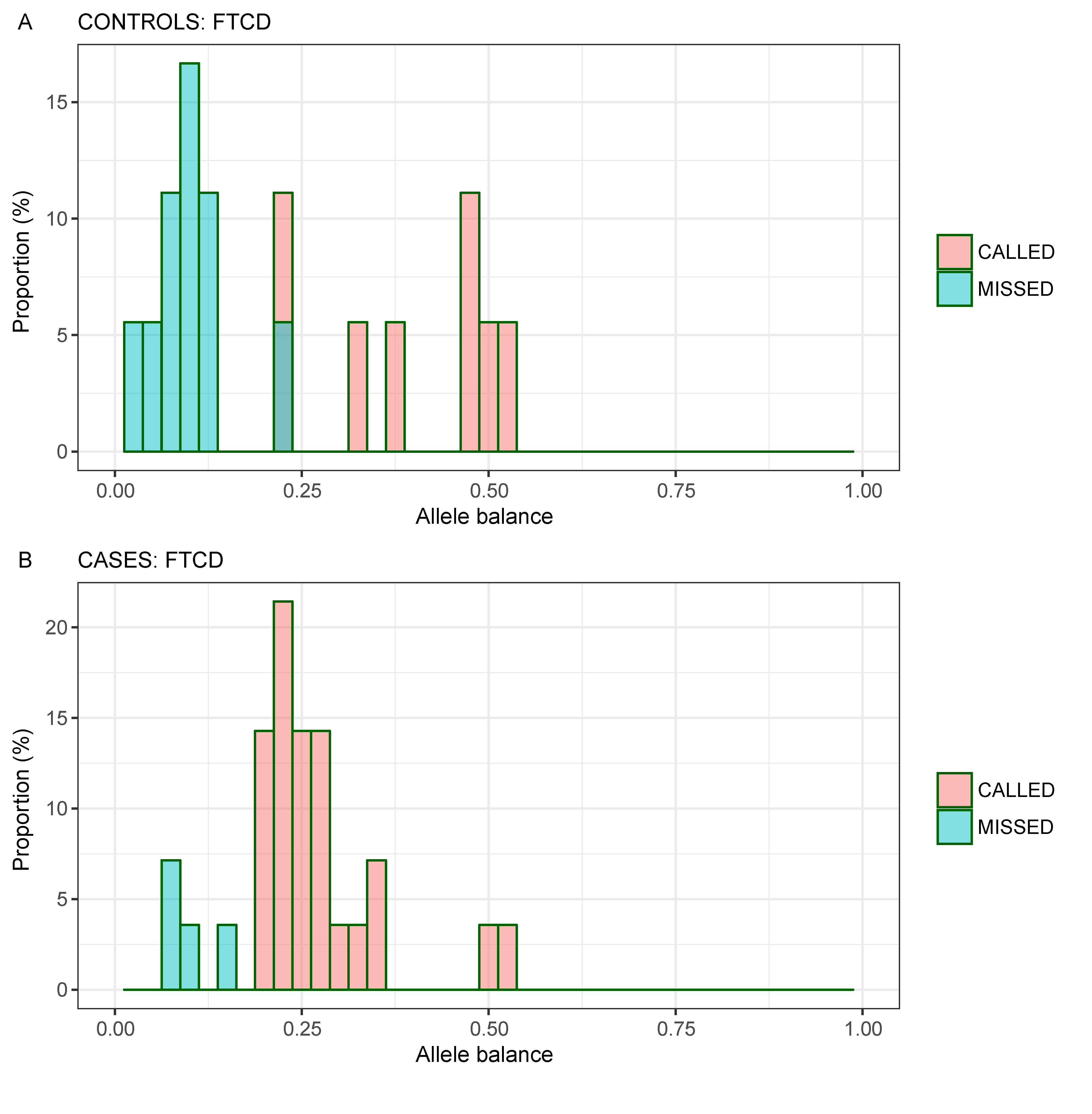

Supplement: S8 Fig — The large number of variants found in FTCD in cases or controls that show a deviation from the expected 50:50 allele ratio expected for heterozygous SNVs, and the different distribution in cases and controls indicate a large number of false positive calls, leading to false gene-phenotype associations. This phenomenon is often caused by un-annotated segmental or tandem duplications in the reference genome, simple sequence repeats or copy gains in the samples. Using the method ABB we identified and excluded these genes from the RVAS test with the ICGC-CLL cohort. (TIF) [file pcbi.1007784.s008.tif]
