# Supplementary material for: Efficient and flexible Integration of variant characteristics in rare variant association studies using integrated nested Laplace approximation
Source: PLoS Comput Biol. 2021 Feb 19;17(2):e1007784. doi: 10.1371/journal.pcbi.1007784 (PMC7928502; doi:10.1371/journal.pcbi.1007784)
Supplement: S1 Table — (DOCX) [file pcbi.1007784.s009.docx]

**S1 Table** BRCA risk variants in ClinVar used for simulation as introduced causal variants.

| **Gene** | **# of candidate variants** | **# of indels** | **# of splicing variants** | **# of stopgain SNVs** | **# of nonsynonymous SNVs** |
| --- | --- | --- | --- | --- | --- |
| BRCA2 | 1213 | 825 | 44 | 283 | 61 |
| BRCA1 | 1037 | 656 | 65 | 262 | 54 |
| PALB2 | 49 | 31 | 0 | 15 | 3 |
| BRIP1 | 25 | 11 | 1 | 9 | 4 |
| CHEK2 | 17 | 8 | 1 | 7 | 1 |
| BARD1 | 7 | 1 | 0 | 5 | 1 |
