# Supplementary material for: Efficient and flexible Integration of variant characteristics in rare variant association studies using integrated nested Laplace approximation
Source: PLoS Comput Biol. 2021 Feb 19;17(2):e1007784. doi: 10.1371/journal.pcbi.1007784 (PMC7928502; doi:10.1371/journal.pcbi.1007784)
Supplement: S2 Table — (DOCX) [file pcbi.1007784.s010.docx]

**S2 Table** Number of variants in six BRCA risk genes in the 1000GP cohort before introduction of risk variants from ClinVar and HGMD (counting only rare coding or splicing variants with CADD > 10).

| **Gene** | **Total # of mutations** | **# of possible cases** | **# of possible controls** | **# of uniq. mutations cases** | **# of uniq. mutations controls** | **# of affected cases** | **# of affected controls** | **# of likely pathogenic mutations in cases/controls** |
| --- | --- | --- | --- | --- | --- | --- | --- | --- |
| BRCA2 | 45 | 898 | 912 | 21 | 31 | 33 | 43 | 6/3 |
| BRCA1 | 26 | 898 | 912 | 18 | 15 | 32 | 18 | 2/1 |
| PALB2 | 26 | 898 | 912 | 17 | 15 | 22 | 17 | 0/0 |
| BRIP1 | 16 | 898 | 912 | 14 | 7 | 20 | 11 | 2/2 |
| CHEK2 | 10 | 898 | 912 | 3 | 10 | 9 | 12 | 0/0 |
| BARD1 | 18 | 898 | 912 | 12 | 11 | 17 | 18 | 2/2 |
