# Supplementary material for: Efficient and flexible Integration of variant characteristics in rare variant association studies using integrated nested Laplace approximation
Source: PLoS Comput Biol. 2021 Feb 19;17(2):e1007784. doi: 10.1371/journal.pcbi.1007784 (PMC7928502; doi:10.1371/journal.pcbi.1007784)
Supplement: S3 Table — Due to less than 100% variant call rates in some positions the number of possible cases and/or controls can be lower than total number of cases (389) and controls (778) used in the Iberian cohort. (DOCX) [file pcbi.1007784.s011.docx]

**S3 Table** Number of variants in six BRCA risk genes in the Iberian cohort before introduction of risk variants from ClinVar (counting only rare coding or splicing variants with CADD > 10). Due to less than 100% variant call rates in some positions the number of possible cases and/or controls can be lower than total number of cases (389) and controls (778) used in the Iberian cohort.

| **Gene** | **Total # of mutations** | **# of possible cases** | **# of possible controls** | **# of uniq. mutations cases** | **# of uniq. mutations controls** | **# of affected cases** | **# of affected controls** | **# of likely pathogenic mutations in cases/controls** |
| --- | --- | --- | --- | --- | --- | --- | --- | --- |
| BRCA2 | 54 | 387 | 776 | 21 | 42 | 30 | 60 | 1/6 |
| BRCA1 | 30 | 389 | 777 | 16 | 20 | 24 | 37 | 1/2 |
| PALB2 | 14 | 386 | 773 | 6 | 12 | 10 | 16 | 0/0 |
| BRIP1 | 17 | 386 | 776 | 8 | 12 | 15 | 17 | 0/0 |
| CHEK2 | 14 | 389 | 778 | 6 | 11 | 7 | 14 | 0/0 |
| BARD1 | 9 | 389 | 778 | 3 | 7 | 4 | 15 | 2/6 |
