# Supplementary material for: Efficient and flexible Integration of variant characteristics in rare variant association studies using integrated nested Laplace approximation
Source: PLoS Comput Biol. 2021 Feb 19;17(2):e1007784. doi: 10.1371/journal.pcbi.1007784 (PMC7928502; doi:10.1371/journal.pcbi.1007784)
Supplement: S4 Table — We randomly permuted case and control labels 10 times and for each estimated empirical thresholds for each RVAS test. The median TIER values from 10 random permutations are used as thresholds for benchmark comparison. (DOCX) [file pcbi.1007784.s012.docx]

**S4 Table** P-value, Bayes Factor (HBMR) and ΔDIC (BATI) thresholds for Type I error rates (TIER) of 0.05, 0.001 and 1e-04 estimated on Iberian cohort. We randomly permuted case and control labels 10 times and for each estimated empirical thresholds for each RVAS test. The median TIER values from 10 random permutations are used as thresholds for benchmark comparison.

| **Method** | **0.05 TIER** | **0.001 TIER** | **1e-04 TIER** |
| --- | --- | --- | --- |
| BURDEN | 5.392e-02 | 7.984e-04 | 9.602e-05 |
| KBAC | 7.229e-02 | 1.520e-03 | 1.813e-04 |
| SKAT-O | 5.834e-02 | 1.265e-03 | 2.400e-04 |
| MiST | 8.077e-02 | 1.160e-14 | 1.110e-16 |
| HBMR | 1.2282 | 3.8680 | 10.6012 |
| BATI | 2.7385 | 12.6450 | 18.3837 |
