# Supplementary material for: Efficient and flexible Integration of variant characteristics in rare variant association studies using integrated nested Laplace approximation
Source: PLoS Comput Biol. 2021 Feb 19;17(2):e1007784. doi: 10.1371/journal.pcbi.1007784 (PMC7928502; doi:10.1371/journal.pcbi.1007784)
Supplement: S5 Table — (DOCX) [file pcbi.1007784.s013.docx]

**S5 Table** Variant positions labelled as potential systematic errors by ABB.

| **Chromosome** | **Position** | **ABB** | **Missed-called-ratio(FDR)** | **Gene** |
| --- | --- | --- | --- | --- |
| 17 | 45232102 | 0.9607 | 1.0911E-16 | CDC27 |
| 17 | 45232109 | 0.9587 | 3.3355E-11 | CDC27 |
| 21 | 47557241 | 0.8790 | 1.6543E-02 | FTCD |
| 17 | 45247305 | NA | 4.3332E-02 | CDC27 |
| 9 | 138517978 | 0.8729 | 4.3332E-02 | GLT6D1 |
