# Supplementary material for: Efficient and flexible Integration of variant characteristics in rare variant association studies using integrated nested Laplace approximation
Source: PLoS Comput Biol. 2021 Feb 19;17(2):e1007784. doi: 10.1371/journal.pcbi.1007784 (PMC7928502; doi:10.1371/journal.pcbi.1007784)
Supplement: S6 Table — (DOCX) [file pcbi.1007784.s014.docx]

**S6 Table** Potential false positive candidate genes.

| **Gene** | **Missed-called-ratio (FDR)** | **Association-ABB (FDR)** |
| --- | --- | --- |
| CDC27 | 4.4310E-19 | 3.2492E-07 |
| FTCD | 4.5453E-02 | 9.4176E-01 |
| GLT6D1 | 4.0541E-03 | 1.6931E-01 |
