# Supplementary material for: Efficient and flexible Integration of variant characteristics in rare variant association studies using integrated nested Laplace approximation
Source: PLoS Comput Biol. 2021 Feb 19;17(2):e1007784. doi: 10.1371/journal.pcbi.1007784 (PMC7928502; doi:10.1371/journal.pcbi.1007784)
Supplement: S7 Table — Likely false positive gene associations labelled by ABB have been removed. (DOCX) [file pcbi.1007784.s015.docx]

**S7 Table** Significant risk genes for CLL detected by BATI, with support of up to 5 other RVAS tests. Likely false positive gene associations labelled by ABB have been removed.

| Gene | Variant positions in cohort | Affected cases | Affected controls | Total cases | Total controls | BATI | HBMR | BURDEN | SKATO | KBAC | MiST |
| --- | --- | --- | --- | --- | --- | --- | --- | --- | --- | --- | --- |
| ECEL1 | 9 | 42 | 21 | 429 | 718 | 60.03 | Inf | 3.54E-07 | 2.81E-12 | 1.00E-06 | 2.15E-11 |
| EHMT2 | 11 | 42 | 22 | 429 | 718 | 43.02 | Inf | 5.17E-07 | 1.33E-10 | 2.00E-06 | NA |
| COPS7A | 4 | 20 | 4 | 432 | 724 | 31.80 | Inf | 7.82E-07 | 4.81E-09 | 1.00E-06 | 1.59E-11 |
| C1QTNF7 | 5 | 19 | 3 | 435 | 725 | 28.18 | Inf | 1.21E-06 | 1.75E-07 | 1.00E-06 | 2.32E-08 |
| ADCY2 | 7 | 24 | 7 | 434 | 723 | 26.26 | 394.83 | 7.26E-06 | 2.05E-06 | 3.00E-06 | 0.000105461 |
| SERAC1 | 9 | 12 | 2 | 436 | 724 | 16.14 | 1.48 | 1.99E-05 | 8.40E-05 | 0.000249998 | NA |
| BCAS1 | 11 | 25 | 15 | 436 | 725 | 15.98 | 2.38 | 0.000518987 | 0.001251352 | 0.000666656 | 0.002687874 |
| HERC6 | 7 | 22 | 11 | 436 | 723 | 15.46 | 1.92 | 0.000247701 | 0.000527661 | 0.000542849 | NA |
| SIRPB2 | 5 | 11 | 2 | 436 | 725 | 15.27 | 1.66 | 0.000152165 | 0.000320107 | 0.000487494 | NA |
| TOMM70A | 10 | 15 | 8 | 433 | 725 | 15.08 | 19.39 | 0.003494764 | 0.000190399 | 0.002299885 | NA |
| SLC6A18 | 10 | 16 | 7 | 432 | 718 | 15.06 | 87.79 | 0.00356925 | 2.85E-05 | 0.000924977 | NA |
| CASR | 10 | 16 | 6 | 432 | 724 | 14.73 | 165.67 | 0.000145954 | 3.59E-06 | 0.000123809 | 4.15E-06 |
| C8orf46 | 3 | 13 | 4 | 432 | 723 | 14.53 | 29.84 | 0.001279714 | 9.40E-05 | 0.000211427 | 7.41E-05 |
| FSTL5 | 14 | 17 | 9 | 435 | 722 | 12.88 | 2.46 | 0.000585093 | 0.001398403 | 0.001599936 | 0.002743657 |
| LAMP2 | 3 | 13 | 5 | 436 | 725 | 12.65 | 2.10 | 0.000887406 | 0.001855312 | 0.000949976 | 0.003353999 |
| DOCK1 | 21 | 18 | 10 | 436 | 722 | 12.65 | 1.15 | 0.000883377 | 0.007279736 | 0.002733151 | 0.005068837 |
| MAP4K2 | 6 | 12 | 4 | 432 | 722 | 12.31 | 1.65 | 0.00056233 | 0.001468045 | 0.001799928 | 0.007891828 |
