# Supplementary material for: Efficient and flexible Integration of variant characteristics in rare variant association studies using integrated nested Laplace approximation
Source: PLoS Comput Biol. 2021 Feb 19;17(2):e1007784. doi: 10.1371/journal.pcbi.1007784 (PMC7928502; doi:10.1371/journal.pcbi.1007784)
Supplement: S8 Table — A total of 16676 genes were tested using an allele frequency threshold of AF< = 0.01 for all identified SNVs and indels. (DOCX) [file pcbi.1007784.s016.docx]

**S8 Table** Run times of six RVAS methods (hours: minutes: seconds) using 12 cores for 898 cases and 912 controls randomly selected from the 1000GP cohort. A total of 16676 genes were tested using an allele frequency threshold of AF<=0.01 for all identified SNVs and indels.

| Method | Burden | KBAC | SKAT-O | MIST | HBMR | BATI |
| --- | --- | --- | --- | --- | --- | --- |
| Runtime (12 cores) | 01:57:56 | 03:10:25 | 03:21:06 | 02:40:11 | 47:33:23 | 67:29:08 |
