# Supplementary material for: Efficient and flexible Integration of variant characteristics in rare variant association studies using integrated nested Laplace approximation
Source: PLoS Comput Biol. 2021 Feb 19;17(2):e1007784. doi: 10.1371/journal.pcbi.1007784 (PMC7928502; doi:10.1371/journal.pcbi.1007784)
Supplement: S1 Text — Provides additional details about a) the cohort simulation method, b) the integration of third-party rare variant association methods in rvGWAS, c) quality control analysis with rvGWAS, and d) evaluation of potential false positive rare variant calls detected within the chronic lymphocytic leukemia risk genes analysis. (DOCX) [file pcbi.1007784.s017.docx]

Supporting information

**Simulating disease variants**.

*Algorithm for determining relative risk (RR) for each breast cancer risk variant*

1. MAF of introduced variants is estimated using the ExAC database. We define minimum MAF as 1e-10 to avoid zeros caused by limited size of ExAC cohort.

1. To take into account the expected exponential decay in RR variability based on the MAF range of values, we used the probability density function beta, that is a family of continuous probability distributions defined on the interval [0, 1], the range of values that MAF can take, and parameterized by two positive shape parameters. The beta distribution with shape parameters α = 0.005 and β = 1 best emulates the expected behavior; therefore we generated quantiles from this beta distribution to obtain reasonable values of RR variability across the range of MAF values. As the resolution for very rare variants (MAF<5e-04) is limited, a minimum between the computed value and 10 will be taken as standard deviation in the next step (Figure S2).
2. For variants with different MAF values we sample RR from a normal distribution with mean equal to zero and standard deviation calculated as described in step 2. In case of the 1000GP cohort add 1.5 to the absolute value, as we have twice as many controls as cases, and minimum RR should be above 1 (not necessary for Iberian cohort with case to control ratio of 1:1). As final relative risk (RR) we take the minimum between the computed value and 16, as we want to avoid extreme scenarios such as a single risk variant affecting 10% or more cases.

Using the RR generation algorithm results in an RR distribution in which risk variants with MAF≈0.01 have RR around 2, while for very rare risk variants (MAF≈0.001) RRs between 1.5 and 16 are generated. The RR distribution is shown in Figure S2. Given the case-control type of design, we have used the odds ratio (OR) instead of RR. Since the prevalence of the simulated disease is very small, the OR is shown to be a good approximation of the RR. The population frequency of the risk allele can simply be estimated from the cohort.

*Algorithm to determine cumulative variance explained (VE) of the phenotype.*

We assumed independence between risk variants at a given locus, and estimated the total percentage of VE as the sum of the VE by each individual variant. The following procedure was used for introducing risk variants for each of the eight simulated breast cancer risk genes:

1. Pick randomly a variant from the pool of the ClinVar candidate variants for a gene.
2. Estimate MAF for this variant with ExAC database.
3. Introduce an effect by sampling from the generated RR distribution.
4. With MAF from (2) estimate number of affected controls. With a number of affected controls and sampled RR from (3) estimate number of affected cases.
5. If number of affected cases and controls is 0 go back to step (1).
6. Introduce variant into the estimated number of cases and controls by sampling randomly cases and controls.
7. Remove the risk variant from the pool of candidate variants for this simulation.
8. Stop if there are no more variants in the pool of candidate variants.
9. If the cumulative variance explained by variants introduced in a gene is below the specified threshold (0.5%, 1% or 2%), go to step (1) and repeat.
10. If the variance is above the specified threshold stop with introducing risk variants

In case of BRCA1 and BRCA2 we additionally evaluated the annotated function of the ClinVar risk variant. We generated two separate architectures for each gene, one only containing missense variants and one only containing non-sense (loss of function or splicing) variants.

**Framework for Rare Variant Genome Wide Association Study (rvGWAS)**

*Quality Control (QC)*

The QC module of rvGWAS implements various quality control procedures. rvGWAS–QC checks the homogeneity of cases and controls and identifies biases in data that could cause high false positive rates. To do so we developed an interactive R script that computes essential quality control (QC) measures and prepares data for association testing. The script requires two mandatory input parameters: 1) sample information file path and 2) variant information file path. The sample information file has only one mandatory column specifying the names of the samples. Additional columns can be used to provide technical and clinical information on each sample, e.g. library preparation type and target enrichment kits, gender, population, etc. The variant information file is similar to multi-sample VCF (variant call format) files with a few changes facilitating functional annotation of variants. A header line is used for specifying the content of columns, where column names that are associated with variant position (inherited from VCF file) and functional annotation start with # and only column names that represents sample’s genotype starts without #. Another difference to VCF is that sample genotypes are annotated as: NA (no call available for the sample), 0 (both alleles called as reference), 1 (heterozygous variant) and 2 (homozygous variant). All columns are tab separated and the following annotation columns are mandatory: #Chr, #Position, #Reference, and #Alteration. Sample names in the header have to match the names given in the sample information file. (If this is not the case there is an optional parameter allowing to specify a translation file with two columns. The first column represents the names of the samples from the variant information file and the second column is the corresponding sample name in the sample information file.)

Users can provide a separate fiIes for SNVs and indels, or integrate them in one file. If separated, the InDel file should have the same format as the genetic variant file used for SNVs. SNV and InDel files will be joined prior to association analysis, but an additional column will be added indicating if a variant is a SNV or an InDel.

The QC script is interactive and offers the user to perform the following steps:

1. Users will be offered to remove any samples based on any column from the sample information file. For example, the user can choose to remove all samples that in the ‘Population’ column have an entry different from ‘Spanish’.
2. Next, users can remove variants based on any annotation column. If an annotation column is categorical users can specify specific entries to keep or remove, e.g. remove anything other than ‘exonic’ or ‘splicing’. If an annotation column is numerical users will be briefly informed on quantiles for the column and can then specify thresholds for minimum or maximum values. An example for such column is ‘#Segmental duplication’, where one can for instance filter out all variants that have 0.95 or higher value.
3. If for some variants genotypes were not called (NAs) users will be offered to remove variants based on percentage of NAs in the cohort (i.e. a minimum variant call rate can be specified).
4. A histogram of number of variants per sample is presented (Figure 1E and Figure S5E). Samples that have a number of variants outside of the limits specified by the user will be removed.

1. A histogram of transition to transversion (Ti/Tv) ratio per sample is presented. Samples that have a Ti/Tv ratio outside of the limits specified by the user will be removed.
2. Users can choose the type of variants for calculating a Principal Component Analysis (PCA). Given large enough variant sets, synonymous SNVs are recommended for PCA calculation as they are considered neutral (i.e. they are very likely not under selective pressure and have no effect on the diseases). In addition, very rare variants (e.g. MAF<0.005) can be removed from PCA, as they are not informative. Furthermore, all variants in linkage disequilibrium (LD ≥ 0.2) are removed from PCA analysis automatically. Optionally, users can choose color and shape for visualization of samples in the PCA projection. For PCA any genotype labeled NA will be replaced with 0, in order to significantly gain in computation speed at the cost of potentially inaccurate projection of samples on PCA components. However, variance explained per component is not expected to change dramatically if variant call rates are high (>80%). The first 20 PCA components will be calculated for the set of selected variants and the percentage of variance explained by each PCA component will be shown to the user (Figure 1F, Figure S5C). Additionally, pairwise projection of the first 10 components will be shown (Figure S5F). Based on these plots the users need to choose the number of PCA components (n) to be included as covariates in association testing.

1. Using parameters determined in the previous steps, PCA components are recalculated, but this time not replacing NA values by 0 (therefore projection on PCA components will be accurate). Numerically this is solved with the Non-linear Iterative Partial Least Squares (NIPALS) [Wold, 1966] function. Samples are projected on the first two PCA components where each sample is colored and given shapes according to previously chosen attributes. Users can choose minimum and maximum values for the first two components where everything outside of chosen limits will be removed.
2. A none-interactive barplot showing the number of variants per sample, colored by variants type/class is generated. Users can choose any annotation column (starting with #) from the variants information file that is categorical to be used as color class. Additionally, users can aggregate and/or rename values from this column (e.g. in column ‘#Exonic Function’ stop gains and stop losses could be renamed to stop gain/loss). An example of such a plot is shown in Figure 1B and Figure S5A.
3. Finally, if the sample information file contains a column specifying cases and controls, additional informative plots will be generated:
   - Barplot of number of mutations, colored by cases and controls. An example of such plot is shown in Figure 1C and Figure S5B. In case one color is biased towards the upper end of the plot we suggest to interrogate why the respective group has inflated number of variants (or the other group deflated numbers). Reasons could range from different populations (biological biases) to different sequencing kits or variant call methods (technical biases).
   - Number of variants per gene in cases versus controls. An example of such plot is shown in Figure 1D and Figure S5D. Here, users can assess if the number of variants per gene is balanced (equally distributed) between cases and controls.

Plots in this step are generated using pre-filtered data obtained in the previous steps and the users can again compare cases and controls on specific types/classes of variants. The output of the QC scripts will contain all plots described above, before and after filtering, if applicable. Additionally, two text files will be generated, one with sample information for all samples passing all filtering steps, as well as n PCA component projections. The second text file is a variant information file with variants passing the all filters. These files are used as input for all RVAS methods. All user choices and any other relevant information from QC are saved in the log file.

*Implementation of various Rare Variant Association Study (RVAS) Methods*

BATI is a novel Bayesian rare variant association test using INLA, which is described in detail in the main manuscript. IT uses the INLA R package version 17.6.20, which offers a user friendly framework and a powerful method for inference on latent Gaussian models. To run the null model as independent variables we use only sample-based covariates, and for the full model we add genetic information. In full model as the latent Gaussian field we use the classical random effects model (by specification of INLA it is equivalent to setting model="z" in formula for random effects). In both models variable family is set to "logistic", variable control.compute to list of values dic=TRUE, cpo=TRUE, config=TRUE, number of threads ("num.threads") to number of available cores specified by user (default 1), and the step length for the gradient calculations for the hyperparameters (h) to value specified by user (default 1e-3). All other parameters are left to default values.

BURDEN is implemented using the package SKAT (cran. r-project.org/web/packages/SKAT/index.html) version 1.3.0. The Null model, which only contains covariates, was generated using the SKAT_Null_Model function with output set to dichotomous outcome (out_type="D") and no sample adjustment (Adjustment=FALSE). All other parameters are set to default. For the actual Burden test with genetic information and covariates we use the function SKATBinary with all parameters at default values except for method, which is set to "Burden" and for weights, for which we use MAF of variants transformed with the Get_Logistic_Weights function with default parameters.

KBAC is implemented using R package (version 0.1) that is available at <https://github.com/gaow/kbac>. This version of KBAC has limitations in parameter options compared to the KBAC standalone software implementation. For example, with the KBAC R package version 0.1 there is no possibility to include covariates. For association testing we use the function KbacTest with parameters alpha=2.5e-06, num.permutation=1000000, with other parameters set to default values.

SKAT-O is implemented in rvGWAS using the package SKAT (same as used for BURDEN test). The Null model, which only contains sample based covariates, is generated using the SKAT_Null_Model function with output set to dichotomous outcome (out_type="D") and no sample adjustment (Adjustment=FALSE). All other parameters are set to default. For SKAT-O association testing with genetic information and covariates we use the function SKATBinary with all default parameters except for method, which is set to "optimal.adj" corresponding to SKAT-O test, and for weights, for which we use MAF of variants transformed with the Get_Logistic_Weights function with default parameters.

MiST is developed as standalone R package and is available as CRAN repository cran.r-project.org/web/packages/MiST/index.html. Here we use we use the function logit.weight.test of the MiST R package version 1.0 with all parameters at default values to perform the association test.

HBMR is implemented using hbmr_bin function from R package BMRV (version 1.32). Parameters used for hbmr_bin function are: (i) pheno - phenotypic vector of zeroes or ones, representing controls and cases, (ii) geno – genotype matrix with 0, 1, and 2 values, (iii) cov – covariate matrix that is first n PCA components in our benchmark (as for other RVAS models when possible), (iv) maf – minor allele frequency vector for variants, (v) the rest of the possible variables are set to default values. Full documentation for the package is available at link <https://rdrr.io/cran/BMRV/>.

**Removal of false positive gene association using ABB**

In order to filter potential false positive associations, we used the method ABB, which detects systematic errors in sequencing data and false positive associations based on the analysis of allele balance bias^1^. The ABB-association evaluation was performed on 20 candidate genes resulting from the RVAS analysis for CLL cases (BATI > 12). ABB labeled 5 out of 170 SNPs as biased sites, meaning that the allele balances of these variants were significantly different between cases and controls (FDR < 0.1; S5 Table column Missed-called-ratio), and shifted from the expected 0.5 for heterozygous SNPs (see Figure S10). In addition, these 5 variants showed a very high ABB scores (average of 0.9178; S5 Table column ABB), indicating a high probability to be systematic variant calling errors^1^. On gene level (aggregated analysis), ABB labeled 3 out of 20 genes as likely false positive associations: CDC27, GLT6D1 and FTCD. The gene-wise test evaluates two criteria. First, the distribution of allele balances between cases and controls, and the ration of called to missed variants was significantly different (FDR < 0.1; S6 Table column Missed-called-ratio) in all three genes. Second, when removing biased sites identified in the position-specific ABB test (see above), associations were not significant anymore for GLT6D1 and FTCD (FDR > 0.1; S6 Table column Association-ABB).

Supplemental References

1. Muyas, F., Bosio, M., Puig, A., Susak, H., Domènech, L., Escaramis, G., Zapata, L., Demidov, G., Estivill, X., Rabionet, R., et al. (2019). Allele balance bias identifies systematic genotyping errors and false disease associations. Hum. Mutat.
