# Supplementary material for: Efficient and flexible Integration of variant characteristics in rare variant association studies using integrated nested Laplace approximation
Source: PLoS Comput Biol. 2021 Feb 19;17(2):e1007784. doi: 10.1371/journal.pcbi.1007784 (PMC7928502; doi:10.1371/journal.pcbi.1007784)
Supplement: Attachment — Submitted filename: Review_comp_Biov2.docx [file pcbi.1007784.s018.docx]

**Review of:**

**Efficient and Flexible Integration of Variant Characteristics in Rare Variant Association**

**Studies Using Integrated Nested Laplace Approximation**

A novel method for rare variant gene-based association analysis, Bayesian rare variant Association Test

using Integrated Nested Laplace Approximation (BATI), is presented here. Through the innovation of employing the numerical (rather than simulation-based) technique Integrated Nested Laplace Approximation (INLA), the method overcomes computational barriers to efficiency, while maintaining the ability to incorporate specific model comparisons to the full model, which improves test performance when compared with other popular methods in a variety of semi-synthetic scenarios. The model allows for the incorporation of variant characteristics in the form of categorical and quantitative variables, and in the manner this feature has been used here, it further enables increases in statistical power. Semi-synthetic genomewide exome sequencing data from the 1000 genomes project and from an in-house collection representing Iberian subjects are used throughout, and genetic architectures of disease are introduced into the samples through the incorporation of rare variants into subjects who were designated as cases. In an applied example, the method allows for novel gene discovery in the context considered. BATI represents a valuable contribution to the field of genetic association studies, addressing an area that has not seen as widespread success as common variant association studies, gene-based rare-variant discovery, in the march toward completing contributions towards disease and trait heritability. By demonstrating high statistical power compared to other commonly used methods, leveraging annotation data on relevant variant characteristics in the setting of the evaluation of full exome sequences with reasonable sample sizes < 2K, this method gives great hope that strides in a variety of applied settings may consequently be made.

In order for the conclusions of the study to be more convincing, one relatively minor presentation point concerning the way variant characteristics are incorporated into the model as well as a more substantive issue concerning the way ethnicity-related population diversity has been taken into consideration, need to be addressed.

**First,** functional variant characteristics:

It is difficult to follow the thread of how individual variant characteristics are considered. The treatment of this point is vague in the methods section. And then further in the manuscript, this becomes more clear. First here is where the treatment is clear:

Lines 354-356: We see that “For BATI and MiST we used prior information on variant characteristics as covariates: CADD scores as a quantitative variable and exonic function (missense, loss-of-function, InDels) as a categorical variable.” It would therefore be extremely useful for this specific example to be generalized and explicated up front and earlier on. It seems like giving flexibility to the future user of BATI on what metrics in classifying variants might be used would be optimal, especially as new algorithms for the prediction of the effects of variants arise continuously.

Particular areas earlier in the text were somewhat obscure:

Lines 168-170:

“BATI can account for individual variant characteristics under the assumption that similar variant-specific characteristics have a similar effect on the function of the protein and hence the phenotype” – What variable in the equations presented holds this information—is this a categorical or quantitative variable?

**Second:** population stratification/ study population diversity:

🡪Lines 274-5: what exactly is meant by “population stratification efficiency”?

🡪“allowing us to benchmark population stratification efficiency of the RVAS tests” what is the measure of efficiency?

The authors interestingly provide one semi-synthetic study source data of mixed ethnicities (IKGP) and another that is more homogeneous in the form of the in-house Iberian cohort. Plots of individuals from the in-house Iberian cohort along PC axes 1🡪3 should be provided in the supplemental materials for an appreciation of this homogeneity. In the 1KGP, ethnicities are adjusted for through the 10 PCs, but there is no stratified analysis strategy. Moreover in the semi-synthetic simulations, ethnicity is not taken into consideration when attributing rare variants to designated cases (Line 288-289: “From 1000GP we randomly selected half of the samples as cases, the other half as controls”). Given that for many diseases, rare underlying variants are ethnicity specific, it would be useful to consider a likely scenario of a distinct rare variant etiology by ethnicity. E.g. could the ClinVar variants be divided among the ethnicities such that each ethnicity receives ethnicity-private variants among cases? This would be one way to address the issue and show the impact on power. Through sampling effects, given that cases and controls are not balanced by ethnicity, there may be scenarios that emerge where an excess of one ethnicity are cases – will this bias results or not? It would seem that other ethnicity specific variants would be identified as false positives. Would matching cases and controls for ethnicity solve potential problems?

Line 421: “However, most tests perform slightly worse due to the smaller size of the cohort.”

How are the authors able to disentangle population heterogeneity effects from sample size effects?

Discussion:

Recommendations should be provided including the use of the deviance information criterion (DIC).

How dependent are the BATI results on the annotations of quantitative measures from CADD? It would be helpful for the authors to comment on the use of other functional categorization algorithms such as Polyphen2. Why would one choose one or another source of annotations in the BATI context?

Is an approach that resembles partitioned heritability possible in the BATI framework? i.e. is it possible to envisage testing the hypothesis that a particular disease is driven by a particular category of variants through the deviance information criterion (DIC). Why or why not?

Line 545: “Furthermore, tests in the original randomized cohorts without introduced disease variants facilitated the translation of method-specific significance thresholds to comparable thresholds for type I error rates.”

Could this sentence be unpacked a bit more – it rests on a number of unexpressed concepts.

Should BATI be used routinely among those interested in common complex disease etiology? Is there a way to extrapolate to the mega biobank scenario with 100K+ sample sizes? What would happen to computational times?

Minor substantive points:

🡪For Figure 1, there needs to be a reworking of the information being conveyed, perhaps by a more accurate and lengthy legend. What is missing is the notion of simulations. The graphs within the figures can only be understood in the context of the semi-synthetic simulations approach that was employed. Different colored cases == different genes?

Lines 207-212

How exactly is the threshold established?

More clarity is needed in this section.

Lines 288-289: What is the rationale for the different balance across cases and controls comparing between the two sources of simulated samples?

Penetrance of the variants:

Lines 299-300:

“As expected, all six genes already had rare variants, likely benign,”

What is the distribution among cases and controls of known breast-cancer causing variants in the 5 genes selected for the simulations? How has penetrance been taken into consideration?

Line 385: “Hence, we removed all genes with p-value 0 from MiST results”

How can this be translated into a recommendation for future users of rvGWAS? It seems like a potential pitfall.

Figure 3

Breast cancer is a condition that has Mendelian-like inheritance, particularly through *BRCA1* and *BRCA2* even though penetrance of most variants is not complete – if the genetic architecture of most other genes underlying common diseases is more similar to *BARD1*, will higher sample sizes help with power?

Para starting line 459 “Only MiST…”. A word about in what way MiST fails would complete the paragraph that starts with MiST and drops it after the first sentence.

Minor presentation points:

Line 98 instead of “hypothesis”, what the author really means is “assumption”

Line 99 “These contemplate…”

Sentence needs to be changed – “these” refers to methods and methods do not contemplate – authors or investigators do.

Line 108. This phrase is too vague: “certain variance that accounts for heterogeneous variant effects.”

Line 178. “allows to construct” 🡪 should be 🡪 “allows for the construction of”

Line 198. Shouldn’t this “Deviance Information Criteria” be “Deviance Information Criterion” instead? (e.g. like Akaike Information Criterion).

Lines 206-207 under null and alternative hypothesis 🡪 should be 🡪 under the null and the alternative hypotheses

Lines 224-5 “allowing to analyze a” 🡪 should be 🡪 “allowing for the analysis of”

Line 243 simplistic should be replaced by simple

Line 251 “are provided in supporting information file.” 🡪 should be 🡪 “are provided in **a** supporting information file.”

Line 253 remove the word “realistic” and instead ensure the explanations below this header fully address the assumptions and to what extent the simulated scenario matches a scenario that might be encountered in a real disease setting. The reader will decide whether the information presented is realistic.

Lines 285-286 The wording is confusing: “The remaining datasets, named 1000GP and Iberian cohort.” It seems you mean “the remaining samples in the two datasets” or something like that?

Line 327: reference genotype – what is the reference? Why not mention the full scope of variation present? Why only those that are non reference? What about overlapping non reference genotypes?

Line 456: Typo – repeat of “BRCA1”: For *BRCA1* and *BRCA1* simulation.

Line 510 at least as good 🡪should be 🡪 at least as well as

Line 514-5 “Therefore, existing tests do not estimate the full model (as in MiST) or use the relatively slow MCMC (as in HBMR)” should instead be something like …existing tests resort to compromises affecting accuracy or remain highly computationally intensive.

Table 1: The P-value, Bayes Factor and deltaDIC labels should be included in the table and not just the title.

Fig 2. Legend. Back to the basics: say what is being depicted – i.e. box plots with IQR even if this is obvious.
