# Supplementary material for: Efficient and flexible Integration of variant characteristics in rare variant association studies using integrated nested Laplace approximation
Source: PLoS Comput Biol. 2021 Feb 19;17(2):e1007784. doi: 10.1371/journal.pcbi.1007784 (PMC7928502; doi:10.1371/journal.pcbi.1007784)
Supplement: Attachment — Submitted filename: Susak_PlosCompBio_2020_Reviewer_response.docx [file pcbi.1007784.s019.docx]

Dear editors of PLOS Computational Biology,

Thank you very much for considering our manuscript for publication in PLOS Computational Biology. We would like to thank the referees and you for the careful assessment of our manuscript. We have carefully evaluated and addressed all suggestions and comments of the reviewers and hope that our responses are satisfactory. With these revisions, we believe that our manuscript has been substantially improved and hope that it is now suitable for publication in PLOS Computational Biology.

Please find below our point-by-point replies to the reviewers’ comments. All additions to the main manuscript and the supplement have been marked in red font. We refer to the changes we have made in the manuscript using consecutive line numbering.

In case you need more information, please do not hesitate to contact us.

Best regards,

Georgia Escaramis on behalf of all authors

**Reviewers' comments:**

**Reviewer #1:** A novel method for rare variant gene-based association analysis, Bayesian rare variant Association Test using Integrated Nested Laplace Approximation (BATI), is presented here. Through the innovation of employing the numerical (rather than simulation-based) technique Integrated Nested Laplace Approximation (INLA), the method overcomes computational barriers to efficiency, while maintaining the ability to incorporate specific model comparisons to the full model, which improves test performance when compared with other popular methods in a variety of semi-synthetic scenarios. The model allows for the incorporation of variant characteristics in the form of categorical and quantitative variables, and in the manner this feature has been used here, it further enables increases in statistical power. Semi-synthetic genomewide exome sequencing data from the 1000 genomes project and from an in-house collection representing Iberian subjects are used throughout, and genetic architectures of disease are introduced into the samples through the incorporation of rare variants into subjects who were designated as cases. In an applied example, the method allows for novel gene discovery in the context considered. BATI represents a valuable contribution to the field of genetic association studies, addressing an area that has not seen as widespread success as common variant association studies, gene-based rare-variant discovery, in the march toward completing contributions towards disease and trait heritability. By demonstrating high statistical power compared to other commonly used methods, leveraging annotation data on relevant variant characteristics in the setting of the evaluation of full exome sequences with reasonable sample sizes < 2K, this method gives great hope that strides in a variety of applied settings may consequently be made.

We would like to thank the reviewer for his/her positive evaluation of our work on for the constructive criticism, we will address in our answers below.

In order for the conclusions of the study to be more convincing, one relatively minor presentation point concerning the way variant characteristics are incorporated into the model as well as a more substantive issue concerning the way ethnicity-related population diversity has been taken into consideration, need to be addressed.

**First,** functional variant characteristics:

It is difficult to follow the thread of how individual variant characteristics are considered. The treatment of this point is vague in the methods section. And then further in the manuscript, this becomes more clear. First here is where the treatment is clear:

Lines 354-356: We see that “For BATI and MiST we used prior information on variant characteristics as covariates: CADD scores as a quantitative variable and exonic function (missense, loss-of-function, InDels) as a categorical variable.” It would therefore be extremely useful for this specific example to be generalized and explicated up front and earlier on. It seems like giving flexibility to the future user of BATI on what metrics in classifying variants might be used would be optimal, especially as new algorithms for the prediction of the effects of variants arise continuously.

Particular areas earlier in the text were somewhat obscure:

Lines 168-170:

“BATI can account for individual variant characteristics under the assumption that similar variant-specific characteristics have a similar effect on the function of the protein and hence the phenotype” – What variable in the equations presented holds this information—is this a categorical or quantitative variable?

Answer: We fully agree and following the reviewer’s suggestion, we now provide examples of the types of variant annotations we consider valuable for improving the performance of tests in lines 172-173). We also provide additional details on how model parameters reflect these variant characteristics and how they are utilized in the association test in lines 177-181).

**Second:** population stratification/ study population diversity:

🡪Lines 274-5: what exactly is meant by “population stratification efficiency”?

🡪“allowing us to benchmark population stratification efficiency of the RVAS tests” what is the measure of efficiency?

A: With population stratification efficiency we meant that the admixed ethnicity dataset allowed us to benchmark the RVAS tests’ performance while having population diversity in cases and controls. By comparing results in the heterogeneous 1000GP cohort to results using the homogeneous Iberian cohort as background, we show that the impact on test results is minimal. As the reviewer points out, we are not benchmarking population stratification efficiency but simply the performance of the RVAS test methods on an admixed ethnicity cohort. We have therefore corrected this statement in the manuscript (lines 286-287) and provide additional information on the ‘ethnicity-aware’ simulation of cases and controls using 1000GP as background in lines 318-324.

The authors interestingly provide one semi-synthetic study source data of mixed ethnicities (IKGP) and another that is more homogeneous in the form of the in-house Iberian cohort. Plots of individuals from the in-house Iberian cohort along PC axes 1🡪3 should be provided in the supplemental materials for an appreciation of this homogeneity.

A: Yes, the results of a principal component analysis for the Iberian cohort are shown in supplemental figure S3F for the first 9 PCA dimensions. However, we missed to reference this figure in the main manuscript. We now added a reference to the figure in lines 289 as follows: “The in-house ‘Iberian’ WES cohort includes 1189 individuals of Spanish ancestry and is highly homogeneous as demonstrated by PCA (Supplementary S3F Fig)”. Moreover, in figure 1F we have added a new inlay panel clearly showing the genetic diversity of the major ethnicities in 1KGP. We thank the reviewer for pointing us at this shortcoming.

In the 1KGP, ethnicities are adjusted for through the 10 PCs, but there is no stratified analysis strategy. Moreover in the semi-synthetic simulations, ethnicity is not taken into consideration when attributing rare variants to designated cases (Line 288-289: “From 1000GP we randomly selected half of the samples as cases, the other half as controls”). Given that for many diseases, rare underlying variants are ethnicity specific, it would be useful to consider a likely scenario of a distinct rare variant etiology by ethnicity. E.g. could the ClinVar variants be divided among the ethnicities such that each ethnicity receives ethnicity-private variants among cases? This would be one way to address the issue and show the impact on power.

A: We would like to thank the reviewer for the guidance she/he has given us in order to properly assess test power in scenarios where rare risk variants may be ethnicity specific. We have simulated the new scenario according to the procedure proposed by the reviewer: samples were separated into populations according to 1000GP annotation and each variant from the pool was assigned to one of the super populations – EUR+AMR, SAS or EAS. Variants annotated in ExAC were assigned to the population with the largest observed allele frequency. Variants not observed in ExAC were assigned to one of the three populations randomly. We describe the new simulation procedure in lines 318-324.

We found that benchmark results on this ‘ethnicity-aware’ simulation only slightly deviate from the results obtained for the original ethnicity-agnostic simulation (see figures below). Nonetheless, we think that the new simulation is more realistic and therefore replaced the original benchmark throughout the manuscript and benchmark figures.

Ethnicity-aware simulation:	Ethnicity-agnostic simulation:

We would like to mention at this point, that in response to reviewer 2 we performed the breast cancer cohort simulations using a much higher prevalence than before. Therefore the presented benchmark results in the manuscript have changed, as we will detail in the answers to reviewer 2.

Through sampling effects, given that cases and controls are not balanced by ethnicity, there may be scenarios that emerge where an excess of one ethnicity are cases – will this bias results or not? It would seem that other ethnicity specific variants would be identified as false positives. Would matching cases and controls for ethnicity solve potential problems?

A: We fully agree with the reviewer that non-ethnicity-matched cases and controls could potentially lead to biased results in scenarios, where an excess of ethnicity-specific private variants are present in combination of an excess of that ethnicity in cases compared to controls (or vice versa). In our simulation this is a minor issue, as random assignment of cases and controls led to a balanced distribution of ethnic groups.

Nonetheless, this bias leaves a minor but observable fingerprint even in our randomized simulation, which is reflected in the analysis of the type I error rates described in lines 386-399 and Supplementary Figure S4 (also see Supplementary S4 Table). Here we performed RVAS tests in 10 independent simulations with randomly assigned cases and controls; and without the inclusion of ClinVar and HGMD risk variants, in order to determine p-value (or BF or DIC) thresholds for reaching a given FDR. In Supplementary Figure S4 one can observe slight fluctuations of the determined p-values especially for the strict FDR of 0.01% (2 false positive genes out of ~20,000). This fluctuation at least partially reflects the differences in random splits of ethnicities to cases and controls in the 10 independent simulations.

Therefore, we suggest avoiding non-matched ethnicities in case-control studies whenever possible independent of the applied RVAS test.

Line 421: “However, most tests perform slightly worse due to the smaller size of the cohort.”

How are the authors able to disentangle population heterogeneity effects from sample size effects?

A: We agree with the reviewer that we cannot disentangle population heterogeneity from sample size effects and we have removed any comment on this line from the manuscript. Interestingly, after increasing disease prevalence for the simulation (as requested by reviewer 2) we did not see this difference anymore.

Discussion:

Recommendations should be provided including the use of the deviance information criterion (DIC).

A: As recommended by the reviewer, we have incorporated in the discussion section choices of the difference in deviance information criterion based on the simulations carried out in our study: “In the case of $\Delta DIC$, a rule of thumb values of $\Delta DIC>10$ are usually recommended to significantly distinguish different models under consideration, here the genetic model against a null model without genetic effects. This is in line with our results obtained from the type I error study in the 1000GP cohort to establish a significance level of 0.1%. In the Iberian cohort, where the sample size is smaller, the specified threshold has been found to be slightly stricter (>12).” (lines 603-607)

How dependent are the BATI results on the annotations of quantitative measures from CADD? It would be helpful for the authors to comment on the use of other functional categorization algorithms such as Polyphen2. Why would one choose one or another source of annotations in the BATI context?

A: We have used CADD because in our experience it performs well for monogenic disorders (we diagnosed more than 6000 index cases using whole-exome sequencing at University Clinics Tübingen). An important factor for chosing CADD for diagnostics was that the score is available for almost all positions of the hg19 genome (or hg38), and we see that as an advantage for RVAS as well. Other meta-predictors such as the more recently developed REVEL or M-CAP that focus on rare variants (both trained on and pre-computed for variants with AF < 1%) outperform CADD, but are only pre-computed for known SNVs (i.e. a problem for novel SNVs). However, BATI allows replacing CADD by any functional impact or evolutionary conservation score or to include multiple scores, and this is a distinguishing feature of our test. We have added a comment on this in discussion section (lines 554-561)

Is an approach that resembles partitioned heritability possible in the BATI framework? i.e. is it possible to envisage testing the hypothesis that a particular disease is driven by a particular category of variants through the deviance information criterion (DIC). Why or why not?

A: We would like to thank the reviewer for pointing us at this interesting option. It is indeed possible within the BATI framework to test if a disease is driven by a particular gene annotation category such as LoF, splicing, indel or missense variants. We can achieve this by comparing model parameter estimates generated by BATI (mean effect and credible interval) as shown in the new Table 3. Using this method one can also compare the impact of different damage scores (if multiple damage scores are used), which would give a hint, which damage score discriminates pathogenic variants better (somewhat comparable to feature selection methods for random forests or other ML methods).

We have added a description of this procedure as a new subsection “Measuring the impact of categorical and numerical variant characteristics” starting at line 498. We demonstrate the feasibility using a scenario where only pathogenic LoF-SNV variants are added to a background of non-pathogenic missense variants. As expected the LoF-SNV category show strongly increased mean effect values, while the missense category shows value close to zero (and credible intervals overlapping zero).

Line 545: “Furthermore, tests in the original randomized cohorts without introduced disease variants facilitated the translation of method-specific significance thresholds to comparable thresholds for type I error rates.”

Could this sentence be unpacked a bit more – it rests on a number of unexpressed concepts.

A: This procedure is described in detail in the paragraph starting in line 386 (Results section). We agree that the quoted sentence from the Discussion is a bit overloaded and hard to understand. We have tried to break it down without simply reiterating the description in the Results part (lines 596-602):

“Furthermore, since the RVAS tests evaluated here use diverse criteria for statistical significance (p-value, Bayes factor or ∆DIC), we generated groups of cases and controls randomly from the 1000GP and Iberian cohorts without adding risk variants for the disease and benchmarked the six RVAS tests. As significant results from such tests can be considered false positives we could establish comparable significance thresholds across the different statistical criteria resulting in similar false positive rates. Hence, we estimated empirically the type I error rate per each RVAS test at different significant levels.”

Should BATI be used routinely among those interested in common complex disease etiology? Is there a way to extrapolate to the mega biobank scenario with 100K+ sample sizes? What would happen to computational times?

A: This is a very interesting question we have contemplated as well. We previously stated in results section:

“The strong performance of BATI in terms of precision and recall comes at the price of longer run time (Supplementary S5 Table). Inference based on full model estimation leads to a higher computational complexity and hence higher run time of BATI compared to all competing methods. The computational time and complexity of RVAS test methods is a concern, as exome and genome sequencing datasets have been increasing dramatically in sample size recently.”

We have moved this paragraph to the Discussion section and added our recommendation on how to extrapolate to a mega biobank scenario: “However, in order to facilitate BATI-based RVAS tests in a ‘mega biobank scenario’ with sample sizes larger than 100K, we recommend to perform a fast RVAS test, such as SKAT-O, to select the top-500 gene candidates and to re-analyze these with BATI to potentially achieve higher power.” (lines 623-635).

Minor substantive points:

🡪For Figure 1, there needs to be a reworking of the information being conveyed, perhaps by a more accurate and lengthy legend. What is missing is the notion of simulations. The graphs within the figures can only be understood in the context of the semi-synthetic simulations approach that was employed. Different colored cases == different genes?

A: We agree that the coloured patients need a proper explanation and that a notion was missing that the workflow is exemplary shown for the simulated breast cancer cohort based on 1000GP exomes. We have added this information.

Lines 207-212

How exactly is the threshold established?

More clarity is needed in this section.

A: We have extended the description as follows: “Finally, we rank the genes by ∆DIC values in ascending order and select a ΔDIC threshold from the quantile corresponding to the desired significance level. For instance, if a significance level of 0.1% is desired, we pick the ∆DIC value of the gene ranked at top 0.1% position as the significance threshold.” (lines 215-218).

Lines 288-289: What is the rationale for the different balance across cases and controls comparing between the two sources of simulated samples?

A: As described in Lewallen S, Courtright P. Epidemiology in practice: Case-control studies. Community Eye Heal J. 1998 <https://www.ncbi.nlm.nih.gov/pmc/articles/PMC1706071/> an important technique for adding power to a case-control study, especially when the number of cases is limited, is to enroll more than one control for every case. It is known, however, that there is little gained by including more than two controls per case. Using exome sequencing controls are often easy to come by, but cases are not. Therefore this is a frequently encountered scenario, which we wanted to interrogate. The Iberian cohort is fairly small, that’s why we decided to go for a 1:2 ratio. We have stated the reasons for this choice in lines 301-304.

Penetrance of the variants:

Lines 299-300:

“As expected, all six genes already had rare variants, likely benign,”

What is the distribution among cases and controls of known breast-cancer causing variants in the 5 genes selected for the simulations? How has penetrance been taken into consideration?

A: We have added this information for the 1000GP cohort in table S2 and the Iberian cohort in table S3. As expected, very few of the pre-existing variants are known breast cancer variants (<= 2 per gene and cohort for most genes). The only exceptions are BRCA2 in both cohorts (not surprisingly as BRCA2 generally shows the most background variants), and BARD1 in the Iberian cohort. For BARD1 the random split led to enrichment of pathogenic variants in the controls (1 vs. 6), which could explain the fairly bad performance of all methods on BARD1. As all methods have to cope with the same benchmark data, we think that the small effect of a few pre-existing risk variants in the benchmark set is negligible.

Line 385: “Hence, we removed all genes with p-value 0 from MiST results”

How can this be translated into a recommendation for future users of rvGWAS? It seems like a potential pitfall.

A: Unfortunately, we cannot offer any good recommendations on how to avoid this issue except for not using MiST on small to medium sized cohorts (unless the error has been fixed in the meantime, but we are not aware of this). The observed issue will likely not affect very large cohorts were ample of variants are found for every gene. Still, we do not want to give negative recommendations and we think we have stated the problem clear enough that users can decide to include MiST, or not.

Figure 3

Breast cancer is a condition that has Mendelian-like inheritance, particularly through *BRCA1* and *BRCA2* even though penetrance of most variants is not complete – if the genetic architecture of most other genes underlying common diseases is more similar to *BARD1*, will higher sample sizes help with power?

A: First we would like to note that phenomena like Mendelian-like inheritance and reduced penetrance are not reflected in our simulation. With that in mind we cannot consider the simulated BRCA1 and BRCA2 risk genes in our cohort to behave exactly like they would in a real breast cancer cohort. We also do not consider if homozygous variants are embryonic lethal, again making the simulated architecture different from the real architecture of BRCA1/2 genetic risk. In summary we can say that we use known risk variants in breast cancer risk genes, but we simulate an ‘average’ disease without adhering to the peculiarities of breast cancer.

Having said that we do believe that larger cohorts will help a lot with power for genes with a genetic architecture like BARD1, for which only few risk variants are known to date and the vast majority of missense variants is benign. But we have no evidence to show for this hypothesis and do not want to discuss it in the manuscript.

Para starting line 459 “Only MiST…”. A word about in what way MiST fails would complete the paragraph that starts with MiST and drops it after the first sentence.

A: We assume there was a problem in the PDF we have submitted, as the sentence seem complete in our Word document. The sentence starting with “Only MiST” in the manuscript is as follows (now line 485):

“Only MiST and BATI are able to leverage categorical variant characteristics, here represented as functional annotations such as ‘missense’, ‘LoF’, ‘indel’.”

Minor presentation points:

Line 98 instead of “hypothesis”, what the author really means is “assumption”

A: Done

Line 99 “These contemplate…”

Sentence needs to be changed – “these” refers to methods and methods do not contemplate – authors or investigators do.

A: We have changed ‘contemplate’ to ‘allow’

Line 108. This phrase is too vague: “certain variance that accounts for heterogeneous variant effects.”

A: We have rephrased this as follows: “variant characteristics and random effects that account for heterogeneous variant effects on phenotype caused by unknown factors” lines 108-109.

Line 178. “allows to construct” 🡪 should be 🡪 “allows for the construction of” done

A: Done

Line 198. Shouldn’t this “Deviance Information Criteria” be “Deviance Information Criterion” instead? (e.g. like Akaike Information Criterion).

A: Yes, we have corrected this.

Lines 206-207 under null and alternative hypothesis 🡪 should be 🡪 under the null and the alternative hypotheses

A: done

Lines 224-5 “allowing to analyze a” 🡪 should be 🡪 “allowing for the analysis of”

A: done

Line 243 simplistic should be replaced by simple

A: done

Line 251 “are provided in supporting information file.” 🡪 should be 🡪 “are provided in **a** supporting information file.”

A: done

Line 253 remove the word “realistic” and instead ensure the explanations below this header fully address the assumptions and to what extent the simulated scenario matches a scenario that might be encountered in a real disease setting. The reader will decide whether the information presented is realistic.

A: we have removed the word realistic

Lines 285-286 The wording is confusing: “The remaining datasets, named 1000GP and Iberian cohort.” It seems you mean “the remaining samples in the two datasets” or something like that?

A: We changed that wording by "the filtered datasets”

Line 327: reference genotype – what is the reference? Why not mention the full scope of variation present? Why only those that are non reference? What about overlapping non reference genotypes?

A: By non-reference genotype we mean any locus that is different from the reference genome hg19. However, we do only consider SNVs and indels, while CNVs, inversions and translocations are ignored. Therefore we now specified the included variant types and the reference genome in lines 350-352:

“Cohorts used for benchmarking of test methods consisted of 1,810 individuals in the 1000GP cohort and 1,167 individuals in the Iberian cohort, harboring 493,314 and 285,658 unique loci, respectively, deviating from the GRCh37 (hg19) reference genome, but only including SNVs and short InDels.”

Line 456: Typo – repeat of “BRCA1”: For *BRCA1* and *BRCA1* simulation.

A: done

Line 510 at least as good 🡪should be 🡪 at least as well as

A: done

Line 514-5 “Therefore, existing tests do not estimate the full model (as in MiST) or use the relatively slow MCMC (as in HBMR)” should instead be something like …existing tests resort to compromises affecting accuracy or remain highly computationally intensive.

A: done

Table 1: The P-value, Bayes Factor and deltaDIC labels should be included in the table and not just the title.

A: done

Fig 2. Legend. Back to the basics: say what is being depicted – i.e. box plots with IQR even if this is obvious.

A: done

**Reviewer #2: Susak et al. propose a novel rare variant burden test to leverage functional annotations of genetic variants. The INLA technique was applied to calculate the posterior. The deviance information criterion was used as a test statistic, and the false positive rate was controlled by permutation.**

We would like to thank the reviewer for his/her careful evaluation of our work on for the constructive criticism, we will address in our answers below.

p.8 line 173-181: Is the model specification used in this study identical to MiST or not? I recommend the authors to clarify this. It's not clear to me whether the method was actually run with a full model that allowed for the dependency structure across variants. It sounds like they did, but I cannot find the details about this in the rest of the manuscript. If the authors ran the full dependency model, the authors need to present data on how good the estimated dependency structures are and whether the algorithm finds a meaningful dependency, in simulations and/or real data. If they are suggesting such a possibility for future studies, I would recommend clarifying this or moving it to discussions. I think the phrase that "this is enabled by INLA" is an over-statement without presenting the data.

A: We would like to thank the reviewer for pointing this out. Indeed, although the method allows running a full dependency model, we have not used this option here. We wanted to mention it solely to show the potential of the method, with the need to be evaluated in future studies. We have clarified this point in lines 184-186.

Benchmark comparisons to MiST: It's great that the new method is more powerful than the previous methods. However, since BATI and MiST are based on similar model specification, it would be great to have some analyses done about why BATI works better with INLA. Is it because of the full dependency structure, what happens if you constrain this to be identical as MiST? Or is the higher accuracy because of calculating full DIC instead of a score test?

A: As answered in previous point, we do not benchmark BATI with correlation structure across variants, so the difference between BATI and MiST must be explainable by differences in model estimation. While MiST constructs a score test under the null hypothesis, BATI uses INLA, which estimates the full likelihood, resulting in higher accuracy and therefore better performance in terms of statistical power. We have added a more detailed explanation to lines 575-578).

p.13 line 309-310: The prevalence of breast cancer is orders of magnitude higher than this. Based on the reference cited, I wonder if the authors perhaps confused incidence rates with prevalence. For the simulation of genetic architecture, the authors need the prevalence (because this is about the liability threshold), not the incidence rate. Simulated architecture with a prevalence of 0.00085 is interesting but unrealistically low not only for breast cancer but also for other typical common complex disorders. I don't think it's appropriate to simulate the allelic heterogeneity of typical common complex diseases under such an extreme prevalence.

We agree and would like to thank the reviewer for pointing us at this problem. The low prevalence used in our original simulation does not represent common complex disorders. We have therefore redone our simulations and all benchmarks based on the breast cancer prevalence 5 years’ period of adult Spanish women (0.66%), defined as the percentage of current cases (new and preexisting) over the specified period of time. This estimate is obtained Global Cancer Observatory website (Ref. Ferlay J, Ervik M, Lam F, Colombet M, Mery L, Piñeros M, Znaor A, Soerjomataram I, Bray F (2018). Global Cancer Observatory: Cancer Today. Lyon, France: International Agency for Research on Cancer. Available from: https://gco.iarc.fr/today/online-analysis-table.

Increasing the prevalence while maintaining the genotype relative risk and minor allele frequency parameters resulted in an increase of variance explained (VE) of the phenotype for each single variant. Therefore, fewer variants than in the original simulation were needed to achieve the desired VE levels (0.5%, 1% and 2%). Hence, the simulation introduced less risk variants into the cohort, which led to a slight drop in power for all benchmarked methods. However, relative performance of the benchmarked RVAS tests remained the same and all conclusions hold. All tables and figures have been updated with the results of the new simulation.

Selection of causal variants: I think it's a great idea to use known ClinVar variants for simulation. However, there could be ascertainment biases in ClinVar variants, i.e. LoFs and missense variants with extreme CADD scores are more likely to be reported to ClinVar. And more subtle variants are less likely to be reported to ClinVar. It would be great if the authors run some of the simulated benchmarks with causal variants sampled from a mixture of both ClinVar and non-ClinVar variants.

A: Following the reviewer’s suggestion we have added additional risk variants from the Human Gene Mutation Database HGMD database (Stenson PD, Mort M, Ball E V., Shaw K, Phillips AD, Cooper DN. The Human Gene Mutation Database: Building a comprehensive mutation repository for clinical and molecular genetics, diagnostic testing and personalized genomic medicine. Human Genetics. 2014, <https://pubmed.ncbi.nlm.nih.gov/24077912/>]) to the simulation pool. A welcome side effect is that we now have enough variants for BARD1 to reliably simulate a VE of 2%, making the gene-wise benchmarking more consistent.

Otherwise we did not observe clear differences in benchmarking results when using additional variants from HGMD. HGMD is compiled based on literature search by experts, while ClinVar is based on user submissions. Therefore, we assume that the inherent biases in these two databases are somewhat different. Nonetheless, it is likely that both DBs preferentially report high damage variants with high CADD score, as these are also preferentially reported in the literature. We were not able to identify an unbiased source of breast cancer risk variants and therefore need to leave this question unanswered for now.
