# Supplementary material for: Efficient and flexible Integration of variant characteristics in rare variant association studies using integrated nested Laplace approximation
Source: PLoS Comput Biol. 2021 Feb 19;17(2):e1007784. doi: 10.1371/journal.pcbi.1007784 (PMC7928502; doi:10.1371/journal.pcbi.1007784)
Supplement: Attachment — Submitted filename: Review_comp_Bio_R1v1.docx [file pcbi.1007784.s020.docx]

**Review of:**

**The revised version (R1) of: Efficient and Flexible Integration of Variant Characteristics in Rare Variant Association Studies Using Integrated Nested Laplace Approximation**

The manuscript has been substantially improved from the previous version. It presents a method, BATI, and software package, rvGWAS, that would be very useful to researchers as they investigate and identify sources of missing heritability for traits of interest.

The authors did well in addressing questions on stratification by ethnicity and functional variant characteristics.

Table 3 was added to the present revised manuscript.

Table 3 BATI (output of the genetic model estimates for the BRCA1-LoF gene architecture

derived from one of the simulations)

--This table shows an interesting result; the demonstration could go a bit further, however. Instead of showing effect sizes only, and that the LoF effect size stands out from the rest, it would be useful to show the deltaDIC scores for both a model that includes the terms distinguishing between the categories of variants (as shown) and one that does not -- therefore allowing for a deltaDIC comparison. One model could include the LoF category alone and another could include a CADD term alone – then the comparison would be the delaDICs across the two and it seems it would become more apparent from an effect identification standpoint what the advantage would be to include separate functional categories.

General writing:

Although it is possible to understand the manuscript as it is written, I would recommend a thorough editing by a professional scientific writer to improve the writing to the level expected for PLoS Computational Biology. See below a few English points I picked up on.

**Abstract:**

Applying rvGWAS to a Chronic Lymphocytic Leukemia study we identified eight

candidate predisposition genes, including *EHMT2* and *COP*

S7A.

Why are these two genes singled out? It would be useful to know how many novel genes for this phenotype (based on the literature) were identified thanks to the novel method, and how many served as positive controls to validate the method.

Discussion

Line 539

and the newly developed BATI test

🡪 the introductory paragraph should recapitulate key features of BATI even if only listing them superficially.

Line 208

the model selection criteria.

Singular so criterion

Line 237

The rvGWAS workflow **is exemplary** shown for a simulated breast cancer

“is exemplary” doesn’t make sense here. Perhaps the authors are saying “is used through demonstrating an example”… not sure. This is a writing point.

Line 247

per principal components should be:

per principal component

Line 433

simulated 8 risk genes 🡪

eight simulated risk genes

Line 547

sore

should be score

Line 601

Appreciated

This statement should include references

Line 610

samples sizes

should be

sample sizes

In the answers to reviewer 2:

We have added a more detailed explanation to lines 575-578).

*When I looked at the lines 575-578, I did not find what I expected: an answer to the question relating to INLA as the difference to explain increased power of BATI vs. MiST. I do not believe the lines pointed to as actually answering the reviewer’s question (it seems that this is a key point – comparing MiST to BATI, is INLA the only difference between the two? – this being the underlying explanation for increased power could be emphasized more).
